# The relation between coping style and posttraumatic growth among patients with breast cancer: A meta-analysis

**DOI:** 10.3389/fpsyg.2022.926383

**Published:** 2022-09-29

**Authors:** Xiao Wan, Haitao Huang, Qianwen Peng, Yiming Zhang, Jiwei Hao, Guangli Lu, Chaoran Chen

**Affiliations:** ^1^College of Nursing and Health, Institute of Nursing and Health, Henan University, Kaifeng, Henan, China; ^2^School of Business, Institute of Business Administration, Henan University, Kaifeng, China

**Keywords:** breast cancer, coping style, posttraumatic growth, meta-analysis, review

## Abstract

Posttraumatic growth (PTG) has been correlated with coping style among patients with breast cancer. However, to date, there is no consensus on the extent to which coping style is associated with PTG in patients with breast cancer. Therefore, we performed a meta-analysis to quantitatively synthesize previous findings. Based on the PRISMA method, this study employed a random effects model using the Stata software (version 16.0) to calculate the pooled correlation coefficient and examined a range of moderators: cancer stage, publication type, participants’ age, and coping style measurement tools. Relevant studies, published from inception to 9 March 2022, were identified through a systematic search in PubMed, Embase, Web of Science, PsycINFO, WANFANG DATA, Chinese National Knowledge Infrastructure (CNKI), and China Science and Technology Journal Database (VIP) databases. Finally, 20 studies involving 3,571 breast cancer patients were included in this investigation. The results showed a high positive relation between confrontation coping and PTG and a moderate positive relation between avoidance coping and PTG (confrontation: *r* = 0.456; avoidance: *r* = 0.291). Additionally, a moderate negative relation was identified between acceptance–resignation coping and PTG (*r* = –0.289). Publication type and coping style measurement tools moderated the relation between coping style and PTG among breast cancer patients. The findings indicated that breast cancer patients should either confront the disease or avoid coping with it according to their disease state, which would facilitate better growth. More studies, especially, large prospective studies, are warranted to verify our findings.

**Systematic review registration:**

[https://www.crd.york.ac.uk/prospero/display_record.php?ID=CRD42022319107], identifier [CRD42022319107].

## Introduction

Breast cancer is amongst the most common malignant tumors in women worldwide (Ghoncheh et al., 2016). According to global cancer statistics, breast cancer was the most commonly diagnosed cancer in 2020, with an incidence rate of 11.7% and a mortality rate of 6.9% (Sung et al., 2021). With the improvement of medical technology, the breast cancer survival rate has gradually increased (Maajani et al., 2019). However, breast loss (mastectomy), surgical chest scars, hair loss, and sexual dysfunction caused by chemotherapy sometimes result in anxiety, depression, despair, fear, etc., which severely impact cancer survivors’ quality of life (Schmid-Büchi et al., 2011; Denieffe et al., 2014; Soriano et al., 2021). In contrast, a few studies have reported that patients with cancer often experience positive psychological changes in the course of their cancer trajectory, known as posttraumatic growth (PTG) (Ýnan and Üstün, 2014; Casellas-Grau et al., 2017; Mostarac and Brajković, 2022).

Posttraumatic growth is defined as the result of an individual’s struggle with a traumatic event (Tedeschi and Calhoun, 2004). Unlike responses to minor or everyday stressors and normal growth and developmental processes (Linley and Joseph, 2004), PTG is an individual’s effort to control the impact of trauma on their lives and attempts to cope with the trauma related experiences and consequences (Tedeschi and Calhoun, 2004). A common used assessment tool is the Posttraumatic Growth Inventory (PTGI) (Tedeschi and Calhoun, 1996), which includes the five dimensions of relating to others, new possibilities, personal strength, spiritual change, and appreciation of life. It comprises 21 items rated on a 6-point Likert-type scale ranging from 0 (I did not experience this change as a result of my crisis) to 5 (I experienced this change to a very great degree). The score ranges from 0 to 105, with high scores indicating positive growth.

In the past few years, many breast cancer patients have reported experiencing PTG (Lelorain et al., 2010; Paredes and Pereira, 2018; Karimzadeh et al., 2021). Furthermore, breast cancer patients have higher PTG levels than healthy people (Bourdon et al., 2019). Researchers have explored potential growth promoters and identified diverse coping styles as a significant psychological adjustment factor to promote faster PTG among breast cancer patients (Buyukasik-Colak et al., 2012; Li et al., 2016; Bellur et al., 2018).

Coping style, or coping strategy, refers to behavioral and cognitive efforts of individuals in response to the environment, adverse life events, or internal needs to manage the internal and external requirements produced by people–situation interactions (Folkman et al., 1986). Feifel et al. (1987) declared that individuals mainly adopt three types of coping methods: confrontation coping, acceptance–resignation coping, and avoidance coping when encountering traumatic events. Among them, confrontation coping is a positive coping style, which implies that the individual is optimistic, rational, non-evasive, prudent, and considers effective methods and strategies to adjust the body and mind to promote health (Kroemeke et al., 2017; Liu, 2018). Acceptance–resignation coping is a negative coping style, which implies that in the face of traumatic events, individuals often feel helpless, disheartened, and negative and lose confidence in treatment and recovery. As for avoidance coping, different research results have different conclusions. A few studies have shown that through avoidance coping style, individuals can divert attention from the disease, suspend conflicts, reduce psychological barriers, and maintain emotional stability to obtain positive results and better quality of life (Ando et al., 2011; Lee et al., 2017); therefore, avoidance coping is considered a positive coping style. However, certain researchers believe that individuals adopt avoidance coping deliberately or even to avoid the impact of traumatic events, which is not conducive to treatment and recovery (Tong et al., 2013); thus, avoidance coping is considered a negative coping mechanism.

Many researchers have investigated the relation between coping style and PTG among patients with breast cancer, and the coping style–PTG relation is still in literature a controversial topic. A few studies have shown a close relation between the two (Ma, 2014; Cheng and Zhang, 2015), several researchers have identified a moderate relation between them (Lisica et al., 2019; Tu et al., 2020), and a few others have suggested a weak relation (Svetina and Nastran, 2012; Romeo et al., 2019). According to Zhang et al. (2017) and Zhao (2021), there is no significant relation between coping style and PTG. One of the reasons for this debate is the small sample size of individual studies; thus, this study used a meta-analysis to integrate previous empirical studies on the relation between coping style and PTG to evaluate the magnitude of the relation between the two factors, providing evidence for whether coping style is associated with PTG.

Additionally, we examined whether the coping style–PTG relation in patients with breast cancer was moderated by certain factors, such as cancer stage, publication type, participants’ age, and coping style measurement tools. First, based on the psychological theory proposed by Janoff-Bulman (2006), patients with advanced cancer experience a greater degree of disruption and psychological stress than those with early stage cancer because of treatment side effects. With the increase in psychological distress, they often adopt a negative coping mechanism, which is not conducive to PTG. Therefore, this relation may vary based on the cancer stage. Second, studies with significant results are more likely to be published, which may tempt authors to exaggerate the true relation between variables (Sterne et al., 2000). To this end, we included dissertations that were not officially published in journals and divided the publication types into two categories, journal and dissertation, to examine the moderating effect of publication type on the relation between coping style and PTG. Third, previous studies have identified that younger breast cancer survivors experience a deeper impact from cancer, reporting greater emotional distress and poorer psychological adjustment than older survivors (Kroenke et al., 2004; Howard-Anderson et al., 2012). Thus, the relation between coping style and PTG in patients with breast cancer may differ based on age. Finally, in terms of the measurement of coping style, the Medical Coping Modes Questionnaire (MCMQ) (Feifel et al., 1987), the abbreviated situational version of the COPE Inventory (Brief COPE) (Carver, 1997), and the Mini-Mental Adaptation to Cancer Scale (Mini-MAC) (Watson et al., 1994) are widely used. As the number of items and dimensions of each scale are different, coping style measurement tools may be a factor that could moderate the relation between coping style and PTG in patients with breast cancer.

To sum up, this study conducted a meta-analysis to deeply investigated the relation between coping style and PTG in patients with breast cancer and examined whether this relation was moderated by (a) cancer stage, (b) publication type, (c) participants’ age, and (d) coping style measurement tools.

## Materials and methods

The current systematic review and meta-analysis was conducted according to the Preferred Reporting Items for Systematic Review and Meta-Analyses (PRISMA) 2020 guidelines (Page et al., 2021). Moreover, the protocol has been registered in PROSPERO (ID: CRD 42022319107)—a prospective international registry of systematic reviews.

### Searching strategy

Relevant literature was retrieved by systematically searching PubMed, Embase, Web of Science, PsycINFO, WANFANG DATA, Chinese National Knowledge Infrastructure (CNKI), and China Science and Technology Journal Database (VIP) databases for studies from inception to 9 March 2022. Search terms used for breast cancer primarily included: “Breast Neoplasms,” “Breast Tumor,” “Breast Tumors,” “Breast Carcinoma,” and “Breast Carcinomas.” Search terms used for PTG primarily included: “Posttraumatic Growth, Psychological,” “Psychological Posttraumatic Growth,” and “Post-traumatic Growth, Psychological.” Search terms employed for coping style mainly included: “Coping Styles,” “Coping Mode,” “Coping Strategy,” “Coping Strategies,” “Coping Behaviors,” and “Coping Skills.” Thereafter, those search terms were combined using appropriate Boolean operators. A detailed search strategy for PubMed is available in the Supplementary material. Furthermore, the reference lists of retrieved articles were manually scrutinized to identify potentially relevant studies.

### Study selection criteria

Two reviewers independently screened the literature, applying the following selection criteria for articles. (1) It reported either the Pearson correlation coefficient *r*, or *F*, *t*, χ^2^, and β values that could be converted to *r* values. (2) PTG measurement instruments were limited to the PTGI or a revised scale based on the PTGI. (3) Coping style measurement tools were limited to scales that could distinguish one or more of the coping strategies of confrontation coping, avoidance coping, and acceptance–resignation coping, such as the MCMQ, the Brief COPE, and the Mini-MAC. (4) Participants were patients with histopathologically diagnosed breast cancer. (5) When duplicate publications reporting on the same participants were identified, the primary study was selected.

The exclusion criteria were (1) articles not written in English or Chinese language; (2) conference reports; (3) low-quality studies as assessed using the 9-item Joanna Briggs Institution Critical Appraisal Checklist (Munn et al., 2015); (4) studies with obvious data errors.

### Data extraction

Two researchers independently collected the data using a purpose-designed form, and in case of disagreements, a consensus was achieved through discussion. We coded the collected studies for the following information: author information, publication year, country, publication type, cancer stages 1–4, participant characteristics, sample size, correlation coefficients between coping style and PTG, and instruments used to measure coping-style level. For the correlation coefficient entry, if studies did not report correlation coefficients *r* but reported *F*, *t*, χ^2^, and β values, according to corresponding formula, they were transformed to *r* values: *r* = $\sqrt{\frac{t^{2}}{t^{2}+d\,f}}$, *r* = $\sqrt{\frac{F}{F+d\,f_{e}}}$, *r* = $\sqrt{\frac{\chi^{2}}{\chi^{2}+N},}$ *r* = β × 0.98 + 0.05 (β ≥ 0); *r* = β × 0.98 – 0.05 (β < 0) [–0.5 < β < 0.5] (Card, 2012).

### Quality appraisal

The methodological quality of all included studies was independently assessed by two researchers using the 9-item Joanna Briggs Institution Critical Appraisal Checklist (Munn et al., 2015). These items are mainly described from nine aspects, including target population, sampling method, sample size, response rate, etc. Relevant details are provided in Supplementary material. “Yes,” “no,” “unclear,” and “not applicable” were the answer options for each item, with 1 point for “yes” and 0 points for the rest. Higher scores reported better methodological quality. Furthermore, any doubt or disagreement was resolved through centralized discussion (among at least three people) or by soliciting the opinions of third-party experts. We considered all included studies to be of moderate to high quality (total score of ≥ 6) (Table 1).

**TABLE 1:** Characteristics of the 20 studies involved in this meta-analysis.

Study author (year)	Country	Publication type	Stage of cancer	Age	N	r			Coping style measurement	JBI score
						
				Mean ± SD		Confrontation	Avoidance	Acceptance–resignation		
Buyukasik-Colak et al., 2012	Turkey	Journal	All stages	45.37 ± 8.72	90	0.403	N/A	N/A	TWCI	6
Svetina and Nastran, 2012	Slovenia	Journal	N/A	61.7 ± 9.7	190	0.224	0.124	N/A	CRI	7
Tong et al., 2013	China	Journal	None-stage 4	45.19 ± 2.53	169	0.358	–0.109	–0.209	MCMQ	7
Ma, 2014	China	Dissertation	None-stage 4	49.87 ± 10.03	300	0.729	0.657	–0.757	MCMQ	8
Cheng and Zhang, 2015	China	Journal	All stages	54.26 ± 11.79	372	0.602	0.495	–0.418	MCMQ	7
Li et al., 2016	China	Journal	None-stage 4	49.9 ± 10	300	0.729	0.657	–0.757	MCMQ	9
Boyle et al., 2017	United States	Journal	None-stage 4	53 ± 8.02	175	0.230	N/A	N/A	Brief COPE	8
Kroemeke et al., 2017	Poland	Journal	N/A	62.27 ± 8.38	84	0.366	0.020	N/A	Brief COPE	7
Tomita et al., 2017	Japan	Journal	All stages	59.08 ± 10.06	157	0.404	N/A	N/A	SCS	7
Zhang et al., 2017	China	Journal	None-stage 4	52 ± N/A	210	0.017	0.121	0.012	MCMQ	7
Bellur et al., 2018	Turkey	Journal	N/A	45.02 ± 8.18	134	0.500	–0.130	0.290	WCI	8
Hu et al., 2018	China	Journal	N/A	55.3 ± 6.6	60	0.604	0.492	–0.416	MCMQ	6
Liu, 2018	China	Dissertation	None-stage 4	48.78 ± 7.56	325	0.592	0.413	–0.581	MCMQ	7
Lisica et al., 2019	Bosnia and Herzegovina	Journal	N/A	55.02 ± 10.03	100	0.352	N/A	N/A	SPC	7
Liu et al., 2019	China	Journal	N/A	45.6 ± 3.4	60	0.790	0.660	0.460	MCMQ	6
Romeo et al., 2019	Italy	Journal	N/A	54.30 ± 8.0	123	0.096	0.124	0.251	Mini-MAC	8
Tu et al., 2020	China	Journal	All stages	51.54 ± 9.7	201	0.460	–0.040	–0.330	Mini-MAC	7
Wang et al., 2020	China	Journal	N/A	N/A	167	0.410	0.259	–0.486	MCMQ	7
Fujimoto and Okamura, 2021	Japan	Journal	All stages	N/A	80	0.353	0.399	N/A	PCI-J	6
Zhao, 2021	China	Journal	N/A	48.21 ± 9.33	274	0.440	0.130	–0.240	MCMQ	8

N/A, Not reported; TWCI, Turkish Ways of Coping Inventory; CRI, Coping Response Inventory; MCMQ, Medical Coping Modes Questionnaire; Brief COPE, abbreviated situational version of the COPE Inventory; SCS, Stress Coping Scale; WCI, Ways of Coping Inventory; SPC, Scale of Proactive Coping; Mini-MAC, Mini-Mental Adaptation to Cancer Scale; PCI-J, Proactive Coping Inventory, Japanese version.

### Statistical analysis

The pooled correlation coefficients and their corresponding 95% confidence intervals (CIs) between PTG and confrontation coping, avoidance coping, and acceptance–resignation coping were calculated using the inverse variance method (Moles, 2009). Specifically, we applied Fisher’s z-transformation to *r*, weighted based on the sample size with 95% CIs: Z=0.5 × ln [(1 + r)/(1 – r)], where the variance of Z is VZ = 1/n – 3, and the standard deviation of Z is SEZ=$\sqrt{\left(1/n-3\right)}$. As suggested by Lipsey and Wilson (2001), effect size *r* values of 0.10, 0.25, and 0.40 correspond to low, moderate, and high correlations, respectively. Heterogeneity across studies was assessed using Cochran’s Q and *I*^2^ statistics (Higgins et al., 2003). A *p* of <0.05 or *I*^2^ of >75% indicated that the between-study heterogeneity was statistically significant. Finally, a random effects model was used to calculate the summary correlation coefficient. Otherwise, the fixed effects model would be used.

Meanwhile, a significant degree of heterogeneity suggested potential moderation effects. The moderating effect analysis involved two forms. (1) When the moderating variable was a continuous variable, we used meta-regression analysis to check whether the result was significant. (2) When the moderating variable was a categorical variable, we used subgroup analysis to test whether the result was significant. Moreover, we performed a sensitivity analysis by sequentially omitting one study for each turn to evaluate the influence of individual studies on the summary correlation coefficients and to test the robustness of the relation between PTG and confrontation coping, avoidance coping, and acceptance–resignation coping. Potential publication bias was detected using funnel plots. Additionally, Begg’s test was performed to help judge publication bias (Begg and Mazumdar, 1994). All statistical analyses were conducted using Stata software (version 16.0).

## Results

### Study characteristics and quality assessment

**FIGURE 1:**
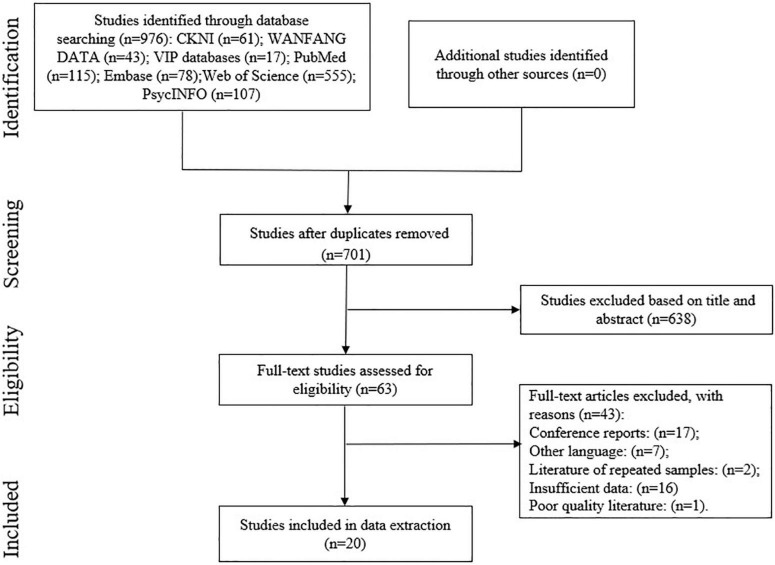
Flow chart of the research selection process.

Our search strategy identified 701 studies without duplicates (Figure 1 is the PRISMA flow diagram of the study screening process). After reading titles and abstracts, full texts of 63 articles were reviewed for eligibility. Of these, 43 studies were excluded because they were conference reports (*n* = 17), in other language (*n* = 7), repeated the samples (*n* = 2), had poor quality (*n* = 1), or had insufficient data (*n* = 16). Finally, 20 studies were included in the meta-analysis, with a total sample size of 3,571 participants. All 20 studies reported correlation coefficients between confrontation coping and PTG. Sixteen studies reported correlation coefficients between avoidance coping and PTG. Thirteen studies reported correlation coefficients between acceptance–resignation coping and PTG. The characteristics of the included studies are summarized in Table 1. The sample size ranged from 60 to 325, and the samples included patients with breast cancer. Among the 20 studies, 2 were from Turkey and Japan each, 11 were form China, and 5 were from Slovenia, the US, Poland, Bosnia and Herzegovina, and Italy each.

### Pooled analyses

As shown in Table 2, random effects models were used for the summary of three different outcomes (heterogeneity for confrontation coping: *I*^2^ = 92.5%, *p* < 0.001; heterogeneity for avoidance coping: *I*^2^ = 94.8%, *p* < 0.001; heterogeneity for acceptance–resignation coping: *I*^2^ = 97.3%, *p* < 0.001). The random effects model showed a high positive relation of 0.456 (95% CI [0.354, 0.548], *p* < 0.001) between confrontation coping and PTG, a moderate positive relation of 0.291 (95% CI [0.139, 0.429], *p* < 0.001) between avoidance coping and PTG, and a moderate negative relation of –0.289 (95% CI [–0.486, –0.064], *p* < 0.001) between acceptance–resignation coping and PTG. Moreover, the relation between coping style and PTG was stable, as the *Z*-value of PTG and confrontation coping, avoidance coping, and acceptance–resignation coping was 7.846 (*p* < 0.001), 3.677 (*p* < 0.001), –2.502 (*p* < 0.05), respectively.

**TABLE 2:** Random effects model of the relation between posttraumatic growth (PTG) and coping styles.

Variable	K	N	Mean *r* effect size	95% CI for *r*	Homogeneity test			Test of null (two tailed)	
					Q(*r*)	*p*	*I* ^2^	*Z*-value	*p*
Confrontation	20	3,571	0.456	[0.354, 0.548]	253.88	0.000	92.5%	7.846	<0.001
Avoidance	16	3,049	0.291	[0.139, 0.429]	288.10	0.000	94.8%	3.677	<0.001
Acceptance–resignation	13	2,695	–0.289	[–0.486, –0.064]	439.32	0.000	97.3%	–2.502	<0.05

### Publication bias and sensitivity analysis

**FIGURE 2:**
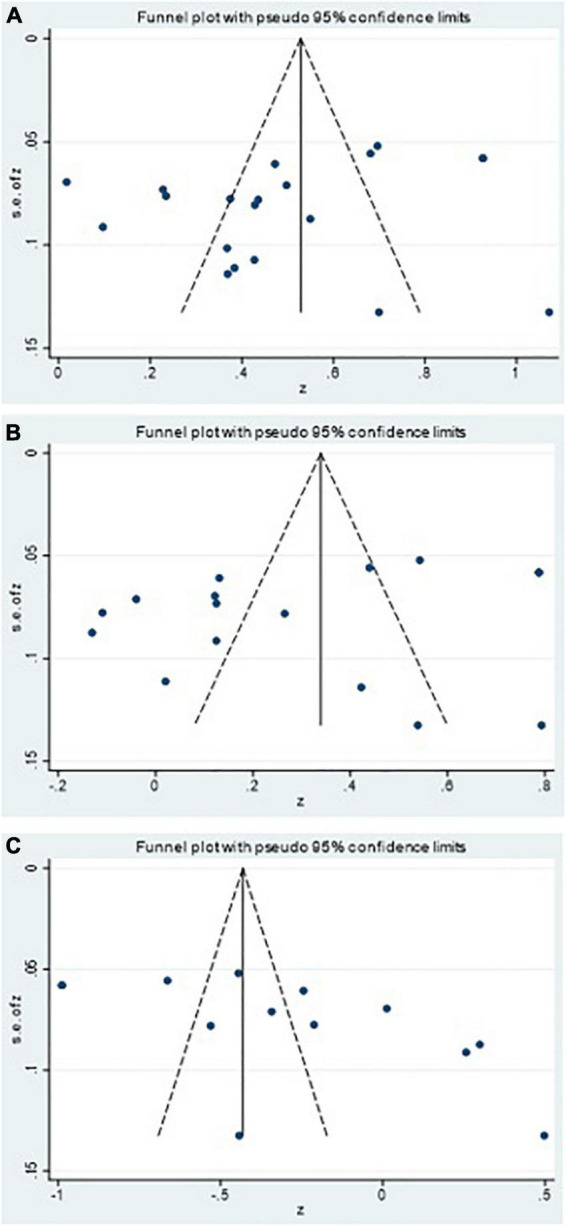
Funnel plots of the relation of posttraumatic growth and **(A)** confrontation, **(B)** avoidance, and **(C)** acceptance-resignation.

First, the meta-analysis was tested by funnel plot for publication bias. Figure 2 shows that the effect sizes of the relation between PTG and confrontation coping, avoidance coping, and acceptance–resignation coping of patients with breast cancer were basically evenly distributed on both sides of the overall effect sizes, implying little publication bias. We used Begg’s test to verify this further. Begg’s rank correlation test also showed little significant bias (confrontation coping: *p* = 0.581; avoidance coping: *p* = 0.892; acceptance–resignation coping: *p* = 0.076). Thus, the published research articles included in this study could systematically and comprehensively represent the research population in this field. Additionally, by sequentially removing one individual study for each turn and then recalculating the summary correlation coefficients, we found that the summary correlation coefficients between coping style (confrontation coping, avoidance coping, and acceptance–resignation coping) and PTG revealed minor changes, suggesting that our results were stable.

### Moderating effect test

In this study, the moderating effect of four variables was tested: cancer stage, publication type, participant’s age, and tool for measuring coping style (Tables 3, 4). For the publication type, the effect of dissertation (confrontation: *r* = 0.666, 95% CI [0.510, 0.779]; acceptance–resignation: *r* = –0.678, 95% CI [–0.816, –0.468]) on PTG and confrontation coping and acceptance–resignation coping among breast cancer patients was significantly larger than that of journal articles (confrontation: *r* = 0.427, 95% CI [0.320, 0.523]; acceptance–resignation: *r* = –0.197, 95% CI [–0.416, 0.043]). Regarding the tool for measuring coping style, the MCMQ had the largest effect on breast cancer patients’ PTG and confrontation coping (*r* = 0.555, 95% CI [0.414, 0.669]) in comparison to the Brief COPE (*r* = 0.280, 95% CI [0.146, 0.403]) and the Mini-MAC (*r* = 0.292, 95% CI [–0.092, 0.600]). The MCMQ (*r* = 0.402, 95% CI [0.228, 0.551]) had a larger effect on breast cancer patients’ avoidance coping and PTG than the Mini-MAC (*r* = 0.032, 95% CI [–0.127, 0.190]). However, the moderating effects of cancer stage and age on coping style and PTG were not significant (all *p* > 0.05).

**TABLE 3:** Coping styles and posttraumatic growth (PTG): Univariate analysis of variance for moderators.

	Between-group effect (QB)	K	N	Mean *r* effect size	95% CI for *r*	Homogeneity test within each group (QW)	*I*^2^ (%)
**Confrontation**							
Cancer stage	0.03						
All stages		5	900	0.462	[0.350, 0.561]	14.56**	72.5
None-stage 4		6	1,479	0.485	[0.226, 0.679]	167.26***	97.0
Publication type	6.31*						
Journal		18	2,946	0.427	[0.320, 0.523]	189.70***	91.0
Dissertation		2	625	0.666	[0.510, 0.779]	9.34**	89.3
CS measurement	8.46*						
MCMQ		10	2,237	0.555	[0.414, 0.669]	167.64***	94.6
Brief COPE		2	259	0.280	[0.146, 0.403]	1.23	18.9
Mini-MAC		2	324	0.292	[–0.092, 0.600]	12.02**	91.7
**Avoidance**							
Cancer stage	0.15						
All stages		3	653	0.298	[–0.089, 0.607]	44.22***	95.5
None-stage 4		5	1,304	0.387	[0.081, 0.626]	140.97***	97.2
Publication type	3.47						
Journal		14	2,424	0.247	[0.086, 0.396]	213.80***	93.9
Dissertation		2	625	0.546	[0.265, 0.742]	18.74***	94.7
CS measurement	9.43**						
MCMQ		10	2,237	0.402	[0.228, 0.551]	185.31***	95.1
Mini-MAC		2	324	0.032	[–0.127, 0.190]	2.03	50.6
**Acceptance–resignation**							
Cancer stage	0.68						
All stages		2	573	–0.385	[–0.465, –0.298]	1.35	26.0
None-stage 4		5	1,304	–0.515	[–0.739, –0.188]	190.92***	97.9
Publication type	9.38**						
Journal		11	2,070	–0.197	[–0.416, 0.043]	301.31***	96.7
Dissertation		2	625	–0.678	[–0.816, –0.468]	16.34***	93.9
CS measurement	1.25						
MCMQ		10	2,237	–0.388	[–0.573, –0.165]	293.67***	96.9
Mini-MAC		2	324	–0.046	[–0.560, 0.494]	26.84***	96.3

*p < 0.05, **p < 0.01, ***p < 0.001.

**TABLE 4:** Univariate regression analysis of year (random effects model).

Variables	K	B	SE	95%CI	*t*	*p*
Confrontation (age)	18	–0.020	0.012	[–0.046, 0.007]	–1.57	0.135
Avoidance (age)	14	–0.006	0.018	[–0.046, 0.033]	–0.36	0.725
Acceptance–resignation (age)	12	–0.033	0.041	[–0.124, 0.058]	–0.80	0.440

## Discussion

### Coping style–posttraumatic growth relation

This systematic review and meta-analyses clarified for the first time the scientific discussion on the magnitude of the relation between confrontation coping and PTG and the magnitude and direction of the relation between PTG and avoidance coping and acceptance–resignation coping. The results showed a highly positive relation between confrontation coping and PTG among breast cancer patients, which was consistent with the findings of most researchers (Tomita et al., 2017; Hu et al., 2018; Liu et al., 2019). It indicated that confronting the disease positively helps boost PTG in patients with breast cancer. The results also showed a moderate positive relation between avoidance coping and PTG in patients with breast cancer, which was in line with the studies by Wang et al. (2020) and Fujimoto and Okamura (2021). It showed that avoidance coping could be seen as a positive coping style, and the process of growth after trauma in patients with breast cancer may require temporary avoidance coping. However, a few studies have suggested that avoidance coping is detrimental to long-term psychological health or attainment of a high level of PTG is unfavorable (Tong et al., 2013; Wilski and Tasiemski, 2016). Thus, more empirical research on the relation between avoidance coping and PTG among breast cancer patients is needed to verify this result. Moreover, the results of our study showed a negative relation between acceptance–resignation coping and PTG in patients with breast cancer, which was consistent with literature (Li et al., 2016; Liu, 2018; Tu et al., 2020). This result indicated that when breast cancer patients adopt acceptance–resignation coping mechanisms to cope with the disease, it is likely to have a negative physical impact. Taken together, these findings demonstrate that coping style is an important variable influencing breast cancer patients’ PTG.

Furthermore, these results showed that PTG was more strongly associated with confrontation and avoidance coping than acceptance–resignation coping was associated with PTG. In other words, positive coping styles are significantly related to breast cancer patients’ PTG. Patients generally do not immediately accept the diagnosis of breast cancer, which is a malignant tumor (Early and Moon, 2021). Thus, they can avoid a sudden mental shock and maintain emotional stability by adopting avoidance coping methods, which divert their attention from the illness (Joseph et al., 2012). However, when patients accept the cancer treatment, numerous avoidance coping methods are adopted, which may be detrimental to the treatment of the disease and the recovery of health. Patients need to actively confront the disease at this time, which can improve their chances of survival and quality of life. Therefore, psychotherapy interventions may focus on coping styles and adopt appropriate and effective coping methods according to the different stages of breast cancer patients to support their PTG.

### Moderating role of cancer stage

For cancer stage, comparing studies based on stages 1–3 patients (non-stage 4 patients) to studies of only stage 4 patients, we identified that the relation between coping style (confrontation coping, avoidance coping, and acceptance–resignation coping) and PTG among breast cancer patients was not moderated by cancer stage, which was inconsistent with our previous hypothesis. This might be explained by the inclusion of an equal number of studies for stage 4 or no–stage 4. Additionally, patients at different stages of breast cancer bear different levels of psychological distress, but there might be patients with similar educational and family environments and personal character traits adopting the same coping methods (Soo and Sherman, 2015; Yeung and Lu, 2018); thus, the difference is not significant.

### Moderating role of publication type

Meta-analyses should include unpublished studies to reduce publication bias (Sterne et al., 2000). Our study included two master’s dissertations not officially published in journals, and the results showed that publication type significantly moderated the relation between PTG and confrontation coping and acceptance–resignation coping among patients with breast cancer. However, the moderating effect on the relation between avoidance coping and PTG was not significant. The correlation coefficients between breast cancer patients’ coping style (confrontation coping and acceptance–resignation coping) and PTG reported in different types of articles were dissimilar, and the degree of correlation reported in dissertations was higher than that reported in journal articles. This difference might be due to the quality of the studies and the rigor of the review. Additionally, as we only included two master’s dissertations, the difference between the number of journal articles and the number of unpublished studies was relatively large, leading to a stronger relation between breast cancer patients’ coping style (confrontation coping and acceptance–resignation coping) and PTG in dissertations.

### Moderating role of age

Meta-regression revealed that the moderating effect of age on the relation between the three coping styles and PTG in breast cancer patients was not significant, which was not consistent with the result of a previous study (Boyle et al., 2017). The reason for this result might be that the majority of the study population we included were middle-aged breast cancer patients, with a small age span. Hence, the relation between the two was not significantly different. Additionally, breast cancer comprises multiple chronic traumas rather than a single event. For different age stages of patients, the diagnosis of cancer may be the trauma or difficult cancer treatment may be the trauma (Tomita et al., 2017). Thus, in an individual case of fighting cancer, breast cancer patients at different age stages may adopt the same coping strategies according to different types of trauma, thereby leading to less variation in the relation between these two variables.

### Moderating role of coping style measurements

The relation between coping style (confrontation coping and avoidance coping) and PTG in patients with breast cancer was moderated by coping style measurement tools. However, the moderating effect on the relation between acceptance-resignation coping and PTG was not significant. The relation between confrontation coping and PTG was higher when the coping style was measured with the MCMQ than with the Mini-MAC and Brief COPE. The relation between avoidance coping and PTG was higher when the coping style was measured with the MCMQ than with the Mini-MAC. The reason for the difference might be that, on the one hand, each scale is divided into different dimensions and items, and on the other hand, the reliability and validity of each scale are different (Cronbach’s α of the MCMQ, Brief COPE, and Mini-MAC are 0.660–0.700, 0.500–0.900, and 0.620–0.870, respectively) (Feifel et al., 1987; Watson et al., 1994; Carver, 1997). Thus, the MCMQ was stable and balanced to a certain extent, whereas the Mini-MAC and Brief COPE had problems, such as unstable and unbalanced factor attribution of certain items. Therefore, the MCMQ can better reflect the relation between breast cancer patients’ coping style (confrontation coping and avoidance coping) and PTG compared to other scales.

### Limitations and prospects

Unlike previous studies on the relation between coping style and PTG among patients with breast cancer, this study adopted the method of meta-analysis to investigate the relation between breast cancer patients’ coping style and PTG, clarifying the controversy about the magnitude and direction of the relation between them in empirical studies. Nevertheless, a few limitations of the current meta-analysis should be considered. First, PTG measurement instruments were restricted to the PTGI or a revised scale based on the PTGI to minimize the potential source of heterogeneity. Similarly, measurement instruments for coping style were restricted to scales that can distinguish one or more of the coping strategies of confrontation coping, avoidance coping, and acceptance–resignation coping. Consequently, only 20 studies were included in the current meta-analysis. Thus, attention should be paid to the interpretation of our findings, as these could be underpowered. Second, given the inclusion of a small number of studies in certain subgroups, the subgroup analyses based on certain moderators should be interpreted with caution. Third, this study focused on the influence of certain moderator variables on the relation between breast cancer patients’ coping style and PTG. Other potential moderator variables, such as gender, time since diagnosis, and PTG measurement tools, should also be analyzed in the future. Fourth, as a few of the included articles did not report effect size directly, and we used a transformed method to calculate the effect size, there might be some bias. Therefore, the search for source material in articles should be expanded in future research.

### Clinical implications

Clinicians and nurses who engage in the assessment and treatment of breast cancer patients and survivors should be aware of these possible relations between coping styles and PTG and understand that each situation is unique. On the one hand, because different stages of breast cancer have different degrees of psychological distress and different psychological problems, clinical medical staff should promote PTG according to the different stages of breast cancer. On the other hand, considering that there are differences in individual characteristics of breast cancer patients, their PTG levels may also be different and affected by various factors. Therefore, clinical medical staff should help breast cancer patients choose appropriate coping methods based on their unique PTG experience and apply them in daily life to promote their physical and mental recovery.

## Conclusion

A significantly positive relation between coping style (confrontation coping and avoidance coping) and PTG and a significantly negative relation between acceptance–resignation coping and PTG have been identified. Both cancer stage and age did not have a moderating effect on the relation between coping style and PTG among patients with breast cancer. Publication type significantly moderated the relation between breast cancer patients’ coping style (confrontation coping and acceptance–resignation coping) and PTG. Coping style measurement tools significantly moderated the relation between breast cancer patients’ coping style (confrontation coping and avoidance coping) and PTG. In the future, more studies, especially, large prospective studies, are warranted to verify our findings.

## Data availability statement

The original contributions presented in this study are included in the article/Supplementary material, further inquiries can be directed to the corresponding authors.

## Author contributions

XW and HH: conceptualization, methodology, formal analysis, and writing—original draft preparation. QP, YZ, and JH: software. XW and GL: validation and data curation. XW and CC: writing—review and editing. GL and CC: supervision. CC: project administration and funding acquisition. All authors contributed to the article and approved the submitted version.
